# Mechanisms of osteopontin and CD44 as metastatic principles in prostate cancer cells

**DOI:** 10.1186/1476-4598-6-18

**Published:** 2007-03-07

**Authors:** Bhavik Desai, Michael J Rogers, Meenakshi A Chellaiah

**Affiliations:** 1 Department of Biomedical Sciences, Dental School, University of Maryland, Baltimore, MD 21201, USA; 2 Bone Research Group, Institute of Medical Sciences, University of Aberdeen, AB252ZD, UK

## Abstract

**Background:**

The expression level of osteopontin correlates with the metastatic potential of several tumors. Osteopontin is a well-characterized ligand for the αvβ3 integrin. The present study was undertaken to elucidate the possible role of osteopontin/αvβ3 signaling in prostate cancer cell migration.

**Results:**

We generated stable prostate cancer cell (PC3) lines that over-express osteopontin (PC3/OPN), mutant OPN in the integrin binding-site (PC3/RGDΔRGA), and null for OPN (PC3/SiRNA). The following observations were made in PC3/OPN cells as compared with PC3 cells: 1) an increase in multinucleated giant cells and RANKL expression; 2) an increase in CD44 surface expression, interaction of CD44/MMP-9 on the cell surface, MMP-9 activity in the conditioned medium, and cell migration; 3) western blot analysis of concentrated conditioned medium exhibited equal levels of MMP-9 protein in all PC3 cells. However, zymography analysis demonstrated that the levels of MMP-9 activity in the conditioned media reflect the CD44 surface expression pattern of the PC3 cell lines; 4) although MMP-9 and MMP-2 are secreted by PC3 cells, only the secretion of MMP-9 is regulated by OPN expression. A strong down regulation of the above-mentioned processes was observed in PC3/OPN (RGA) and PC3/SiRNA cells. PC3/OPN cells treated with bisphosphonate (BP) reproduce the down-regulation observed in PC3/OPN (RGA) and PC3/SiRNA cells.

**Conclusion:**

Rho signaling plays a crucial role in CD44 surface expression. BPs inhibits the mevalonate pathway, which in turn, prevents the prenylation of a number of small GTPases. Attenuation of Rho GTPase activation by BPs may have contributed to the down regulation of cell surface CD44/MMP-9 interaction, MMP-9 activation/secretion, and cell migration. Taken together, these observations suggest that CD44 surface expression is an important event in the activation of MMP-9 and migration of prostate cancer cells. The various steps involved in the above mentioned signaling pathway and/or the molecules regulating the activation of MMP-9 are potential therapeutic target.

## Background

Prostate cancer is a disease of extensive metastases, with secondary lesions occurring in lymph nodes, bones and sometimes in visceral organs, such as the liver, lungs, and even the brain. The advanced stage of prostatic carcinoma eventually metastasizes to the bones in 85–100% of cases. Although metastasis to bone, especially the spine, pelvis, and ribs, is predominantly observed in prostate cancer patients, the mechanism(s) underlying the predilection of prostate cancer to metastasize to bone remains unclear. Chemotactic experiments using extracts from various organs have demonstrated that bone extracts are more potent in attracting prostate cancer cells than other extracts [1]. Some studies have demonstrated an elevated expression of osteopontin (OPN) in highly invasive metastatic breast and prostate cancer cells [2-4]. OPN functions both as a cell attachment and chemoattractive factor in tumors, like breast and prostate cancers [5,6].

OPN interaction with integrin αvβ3 transduces cell-matrix signaling directed to increased motility, invasion, and angiogenesis [7]. Integrin αvβ3 has a role in the metastasis of prostate cancer cells to bone by arbitrating adhesion to and migration on OPN and vitronectin, which are common extra cellular matrix (ECM) proteins in bone microenvironment. Adhesion of breast and prostate cancer cells to bone marrow endothelial cell line (hBMECs) is directly related to the surface expression of the hyaluronan receptor CD44 [8]. *De novo *expression of CD44 and its variant isoforms has been associated with aggressive behavior in various tumors [9]. Also, CD44 expression on prostate cancer cells (PC3) derived from bone metastases has been shown to have a role in their selective adhesion to bone marrow endothelium. PC3 cells exhibited a rapid and strong adhesion to human bone marrow endothelial cell line (hBMECs), and depletion of CD44 expression with use of RNAi attenuated this adhesion [8]. We have reported previously that OPN expression in human melanoma cells increases CD44 surface expression, MMP-2 secretion, and cell migration [10].

Matrix metalloproteinases (MMPs) have been implicated in bone resorption and tumor progression [11]. In many tumor cells, MMPs and CD44 were strongly expressed [12]. The expression of MMPs and variant CD44 (vCD44) correlates strongly with cancer cell invasiveness and metastasis [13,14]. MMPs have a role in tissue remodeling during development, bone resorption, wound healing, and angiogenesis [15-17]. MMP-2 and MMP-9 are associated with metastasis of prostate cancer cells to bone [18]. CD44 associates with a proteolytic form of the matrix metalloproteinase-9 (MMP-9) on the surface of mouse mammary carcinoma and human melanoma cells. CD44 was shown to anchor MMP-9 on the cell surface. Disruption of CD44/MMP-9 cluster formation, by over expression of soluble or truncated cell surface CD44 reduces tumor invasiveness *in vivo *[19].

Several MMP inhibitors have been investigated in clinical trials for their efficacy in blocking tumor invasion. Bisphosphonates (BPs) reduce the rate of damage caused to bone. They inhibit osteoclast activity and, therefore, are used to treat patients with osteolytic metastases. BPs reduce osteoclast bone resorption and pain in prostate cancer patients [20]. Furthermore, oral alendronate therapy protects bone against tumor cell attachment and possibly prevents bone metastases [11]. Bisphosphonate pretreatment of breast and prostate carcinoma cells inhibited tumor cell invasion. These compounds did not induce apoptosis in tumor cells to inhibit tumor invasion, but they inhibited the proteolytic activity of metalloproteinases through chelation of zinc ions [20].

The present study supports the hypothesis that OPN/αvβ3 signaling-mediated CD44/MMP-9 complex formation on the cell surface, MMP-9 secretion, and activity may play important roles in prostate cancer cell migration. A mechanism for the activation and secretion of active MMP-9 has yet to be elucidated. Observations in PC3/SiRNA and PC3/OPN (RGA) cells emphasize the novel role of OPN and αvβ3 signaling pathway in these processes. CD44/MMP-9 complex formation on the cell surface may represent a unique motility-enhancing signal in prostate cancer cells, thereby promoting their invasiveness.

## Results

### Analysis of osteopontin expression in different PC3 clones and the effects of osteopontin expression on MMPs activity in conditioned media

**Figure 1:**
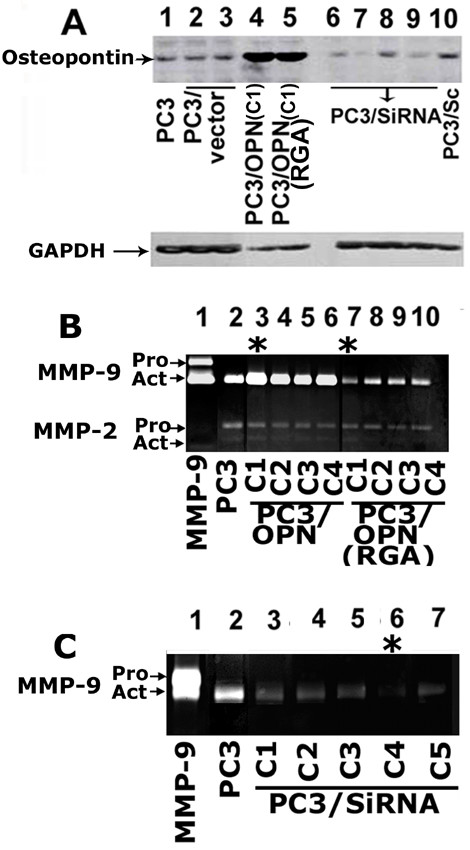
**Analysis of the effects of osteopontin (OPN) expression on MMP-9 activity. *A***. OPN expression levels were measured in different PC3 cell lines (indicated below each lane) by immunoblotting analysis. Untransfected PC3 cells (lane 1), PC3 cells transfected with pCEP4 vector (lane 2), pSilencer 4.1-CMV neo vector (lane 3), and Scrambled RNA construct (lane 10) were used as controls. Basal level expression of OPN was observed in these cells. An increase in OPN expression was observed in PC3 cells transfected with full length (PC3/OPN; lane 4) and mutated (PC3/OPN (RGA); lane 5) OPN cDNA. Individual clones (denoted as C1) that express maximum level of OPN is used for the studies shown here (lanes 4 and 5). PC3 cells transfected with four different SiRNA constructs exhibited expression of different levels of OPN (lanes 6–9). Immunoblotting with an antibody to GAPDH was used as a loading control (lower panel in A). ***B and C: Analysis of MMPs activity in the conditioned media of different PC3 cell lines by gelatin zymography: ***Several individual clones (10–15) were isolated from PC3 cells transfected with different OPN constructs (full length, mutated (RGDΔRGA), and SiRNA). Conditioned media collected from clones that express maximum (Figure B, lanes 3–10; C1–C4 in PC3/OPN and PC3/OPN(RGA)) and reduced levels (Figure C, lanes 3–7, C1–C5) of OPN protein were used for zymogram analysis Untransfected PC3 cells were used as control (lane1 in B and C). The activity of a recombinant MMP-9 protein containing pro- and active band was used as an identification marker (lane 1 in B and C). Gelatinolytic activities of both MMP-2 and MMP-9 are indicated by arrows (B and C). The results represent one of three separate experiments performed.

To determine the OPN/αvβ3-mediated signaling mechanisms involved in prostate cancer cell migration, we generated different PC3 cell lines as described in the Methods section. OPN expression levels were detected by immunoblotting with an antibody to OPN (Figure 1A; top panel. Approximately 10–15 clones were analyzed for high expression of OPN by Western blotting with an antibody to OPN. Among the clones tested, we chose one that exhibited high expression (>10 times) of full length OPN (clone C1; lane 4) and mutant (RGDΔRGA) OPN (Clone C1; lane 5). Untransfected and pCEP-4 vector transfected PC3 cells (lanes 1 and 2) were used as controls. To reduce endogenous OPN in PC3 cells, we generated four different OPN SiRNA constructs in the pSilencer vector (lanes 6–9). Scrambled RNA constructs (lane 10) and vector (pSilencer 4.1-CMV neo vector) -transfected cells (lane 3) were used as controls. A significant decrease in OPN expression was observed in PC3 cells transfected with a SiRNA construct. This construct was targeted to human OPN cDNA sequence 383–403 (lane 7). Immunoblot that is shown in panel A was stripped and blotted with an antibody to GAPDH (Figure 1A, bottom panel) as a loading control.

Several clones (10–15) were isolated from the SiRNA construct targeted to human OPN cDNA sequence 383–403. OPN levels were significantly reduced in five SiRNA clones (C1–C5) as compared with scrambled RNA constructs and vector (pSilencer 4.1-CMV neo vector)-transfected cells. To determine the effects of OPN levels on MMP-9 activity, conditioned media was collected from four independent clones that express high level of full length OPN (B, lanes 3–6), mutant OPN (B; lanes 7–10) and five independent SiRNA clones (C; lanes 3–7). These conditioned media were used for gelatin zymogram analysis (Figure 1B and 1C).

#### Zymogram analysis

OPN functions as a paracrine and autocrine mediator of prostate cancer growth and progression [25]. Cancer cells express high level of MMPs that were shown to assist in tumor initiation, invasion, and tumor metastasis [26]. Therefore, we investigated whether the secreted MMP-9 activity is associated with the OPN expression level. Gelatin zymography exhibited gelatinolytic activity of both MMP-2 and MMP-9 in the conditioned media (Figure 1B). While MMP-2 activity remains the same, changes in MMP-9 activity was observed in PC3 cell lines. MMP-9 activity was greatly enhanced in OPN over expressing clones which are represented as C1–C4 (B, lanes 3–6) as compared with PC3 cells (lane 2). MMP-9 activity is significantly reduced in PC3/OPN (RGA) (B, lanes 7–10) although these individual clones express maximum levels of mutated OPN as shown in Figure 1A (lane 5). PC3/SiRNA clones also exhibited maximum decrease in MMP-9 activity. This corresponds to the OPN expression levels (C, lanes 3–7). Conditioned medium from untransfected PC3 cells (Lane 2 in Figures 1B and 1C) was used as control.

We found an increase in an active form of MMP-9 in PC3 cells. The secretion of this form is greatest in PC3/OPN cells. To further confirm that the secreted MMP-9 is in the active form, we incubated the conditioned medium (10 μg protein) with and without APMA (1 mM at 37°C; 15 min.). In the gelatin zymogram analysis, neither pro- nor an intermittent latent form of MMP-9 was observed after incubation with APMA (data not shown). This confirms that PC3 cells secrete active MMP-9. Although, further experiments are required to fully understand the significance of this observation, it underlies the importance of studying MMP-9 activity in cell migration. Constitutive secretion of active MMP-9 in PC3 cells suggest that the signaling molecule(s) or αvβ3-mediated signal transduction pathway that regulates the activation of MMP-9 may be a potential therapeutic target(s). αvβ3-mediated signal transduction pathway or OPN expression has no significant effect on the already low basal level activity of MMP-2. Asterisks in Figure (1B and 1C) represent the clones used for the present study.

### Analysis of CD44 surface expression and migration in PC3 cell lines

**Figure 2:**
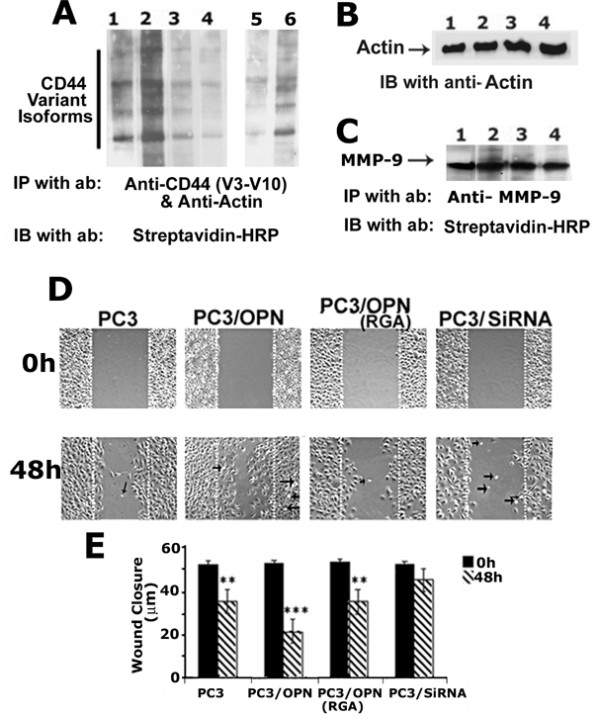
**Analysis of CD44 surface expression and migration in different PC3 cell lines**. **A-C**. Analyses shown in Figures A-C were performed in the following PC3 cell lines: PC3 (lane 1), PC3/OPN (lane 2), PC3/OPN (RGA) (lane 3), and PC3/SiRNA (lane 4). Cells were surface- labeled with NHS-Biotin and lysates were immunoprecipitated with an antibody to vCD44 (V3-10) (A) or MMP-9 (C). Also, as an internal control, a monoclonal antibody to actin was added to vCD44 immunoprecipitation. Actin immunoprecipitation was used as an internal control for normalization. Expression of variant forms of CD44 was observed in PC3 cell lines. Immunoprecipitation with a species-specific non-immune serum did not show any protein bands in the immunoblotting analysis (data not shown). Shorter exposure blot for PC3 (lane 5) and PC3/OPN (lane 6) is shown. The immunoblot shown in A was stripped and blotted with an actin antibody (B). Detection of surface expression of MMP-9 by immunoblotting with streptavidin-HRP is shown in C. No changes in the surface levels of MMP-9 indicate that biotinylation reaction was equally efficient in the indicated PC3 cell lines. The results represent one of three experiments performed. ***D and E: ***Wound healing assay. Phase-contrast micrographs of PC3, PC3/OPN, PC3/OPN (RGA) and PC3/SiRNA cells at 0 h and 48 h are shown. Results represent one of three experiments performed. Statistical analysis is provided as a graph at 0 h and 48 h in panel E. A significant increase in the migration of PC3/OPN, PC3, and PC3/OPN (RGA) cells was observed as compared with PC3/SiRNA cells. *** p < 0.001 and ** p < 0.01 vs. PC3/SiRNA cells. For each cell line, two plates were used per experiment. Multiple uniform streaks (~7–9 streaks) were made on the monolayer culture for each cell line. The data are mean ± SEM of three experiments.

We assessed the surface levels of CD44 in PC3 cell lines. PC3 cell lines were surface labeled with NHS-Biotin and equal amount of protein lysates were immunoprecipitated with an antibody to variant CD44 (vCD44 v3–v10) and actin. Actin antibody was used as an internal control for immunoprecipitation. Immunoblotting was performed with an antibody to streptavidin-HRP to visualize the surface expression of CD44 (Figure 2A). Surface expression of CD44 splice variants (vCD44) was markedly increased in PC3/OPN cells (Figure 2A, lane 2) as compared with PC3 cells (lane 1). Lanes 5 and 6 represent shorter exposure blots of lanes 1 and 2, respectively. Both PC3/OPN (RGA) and PC3/SiRNA cells exhibited a decrease in the surface expression of vCD44 (lanes 3 and 4). The blot shown in the Figure 2B was stripped and immunoblotted with an antibody to actin. Immunoblotting with an antibody to actin did not demonstrate a significant change in the levels (Figure 2B) despite changes in the surface levels of CD44 (Figure 2A). This indicates that equal amount of lysate proteins were used for immunoprecipitation. Our results demonstrate a significant increase in the surface expression of CD44 in PC3/OPN cells.

Since there was an increase in MMP-9 activity in the conditioned media of PC3/OPN cells (Figure 1B), we determine the surface levels of MMP-9 in PC3 cell lines (Figure 2C). Equal amounts of surface labeled lysates were immunoprecipitated with an antibody to MMP-9 and blotted with streptavidin-HRP. There are no changes in the surface levels of MMP-9 in PC3 cell lines, and only a single band of MMP-9 protein (85–90 kDa) was detected in all cells (Figure 2C). No changes in the surface levels of MMP-9 also serve as a biotinylation control for surface labeling. This indicates that the biotinylation reaction was equally efficient in all PC3 cell lines. Consistent with our previous observation [22], the observed increase in the surface level of CD44 is due to OPN/αvβ3 signaling as PC3/OPN (RGA) cells exhibited a decrease in CD44 surface expression.

#### Wound closure assay

Having established that OPN expression increases secretion of MMP-9, we next examined the functional consequences of this in an *in vitro *by migration assay. PC3 cell lines were subjected to wound closure assay (Figures 2D and 2E). The decrease in wound size corresponds to the increase in migration of cells. Migration was performed for 48 h (Figure 2D). Migration of PC3/OPN cells was significantly greater than that of PC3 or PC3/OPN (RGA) cells; migration rates are in the order of PC3/OPN > PC3 = PC3/OPN (RGA) > PC3/SiRNA. Statistical analysis is provided as a graph in Figure 2E. These results suggest that the increase in motility of PC3/OPN cells is due to the interaction of RGD with the αvβ3 integrin, as PC3/OPN (RGA) exhibit basal level migration.

### Analysis of surface interaction of CD44 with MMP-9

**Figure 3:**
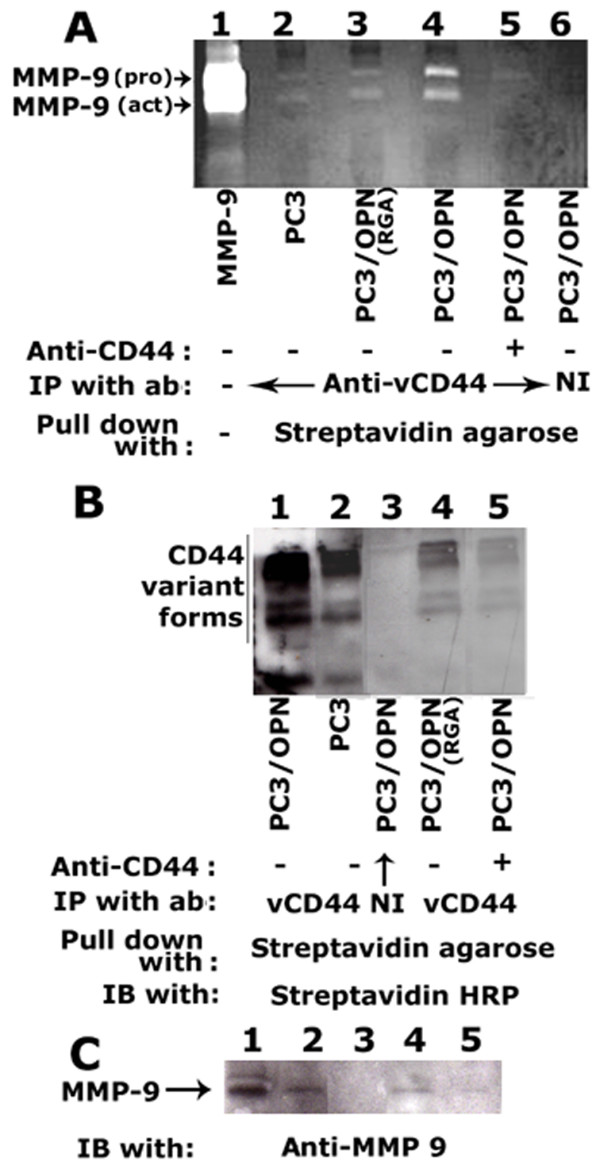
**Analysis of surface interaction of CD44 with MMP-9 in different PC3 cell lines. **Equal amount of proteins were immunoprecipitated with a vCD44 antibody or a non-immune serum as indicated in panels A and B. Immune complexes were subsequently pull-down with streptavidin agarose. A. One half of the immunoprecipitates were analyzed for MMP-9 activity associated with CD44 by zymogram analysis as described in the Methods section. Activity of a recombinant MMP-9 protein was used as an identification marker (lane 1). Arrows indicate pro- and active MMP-9 proteins. B and C. The second half of the immunoprecipitate was blotted with streptavidin HRP to detect surface levels of CD44 (B) and then immunoblotted with anti-MMP-9 (C) after stripping. The results represent one of the three separate experiments performed.

**Figure 4:**
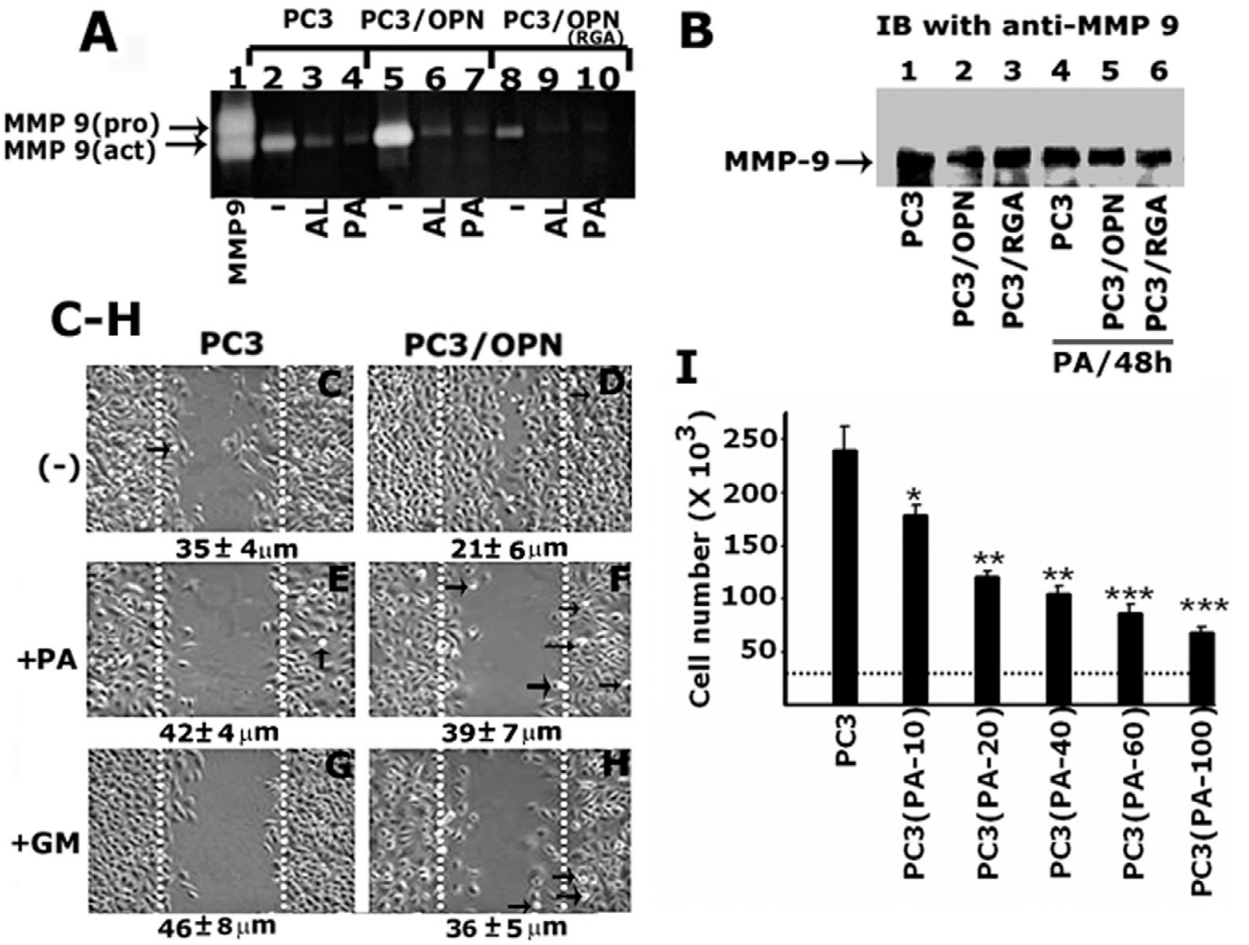
**The effects of bisphosphonates on MMP-9 activity and migration of different PC3 cells. A. **The effect of BPs, such as alendronate (AL, lanes 3, 6, and 9) and pamidronate (PA, lanes 4, 7, and 10) on MMP-9 activity is shown by gelatin zymography. MMP-9 activity was determined in the conditioned media of different PC3 cells indicated in the figure. Untreated cells were used as controls (lanes 2, 5, and 8). The activity of a recombinant MMP-9 protein was used as an identification control. Arrows indicate pro- and active forms of MMP-9. The results represent one of three separate experiments performed. **B. **The effect of BP on the total cellular levels of MMP-9 was determined by immunoblotting with an antibody to MMP-9. No significant changes in the total cellular levels of MMP-9 were observed in response to BP treatment. **C-H. **The effects of pamidronate (PA) and GM6001 (GM) on the migration of PC3 cell lines are shown by a wound closure assay. Statistical analysis is provided at the bottom of each panel as unhealed wound distance (in μm) at 48 h with (+) and without (-) pamidronate (panels E and F) or GM6001 (panels G and H). The data represent the mean ± SEM of triplicate determinations of three different experiments. **I**. The effects of pamidronate on the proliferation of PC3 cells. Dotted line indicates the initial cell number at the time of plating (2.5 × 10^4 ^cells). PC3 cells were treated with 10–100 μM PA for 48 h. A dose dependent decrease in the proliferation was observed in response to PA treatment. Data represent a total of three independent experiments and are expressed as mean ± SEM.

Surface association of CD44 with MMP-9 was shown to provide a mechanism for tumor invasion in mouse mammary carcinoma and human melanoma cells [19]. Expression of antisense CD44 and treatment of cells with anti-CD44 blocked hyaluronic acid (HA)-dependent MMP-2 secretion and, subsequently, invasion of a human lung carcinoma cell line [27]. Given the above observations, which implicate CD44 and MMP-9 in tumor invasion, we sought to determine the surface interaction of these proteins in PC3 cell lines labeled with NHS-Biotin (Figure 3). In order to further corroborate the interaction of MMP-9 with CD44, PC3/OPN cells were also treated with a blocking antibody to CD44 (30 μg/ml; Figure 3, lane 5) prior to labeling with NHS-Biotin. Lysates were immunoprecipitated with a variant CD44 (vCD44) antibody (lanes 2–5) or a non-immune serum (NI, lane 6) and subsequently pulled down with streptavidin agarose. MMP-9 activity associated with the CD44-immunecomplex was analyzed by zymogram analysis as described in the Methods section. Zymogram analysis of one half of the immunoprecipitates is shown in Figure 3A. An increase in CD44-associated MMP- 9 activity was observed on the surface of PC3/OPN cells (Panel A, lane 4). Very minimal activity was observed in PC3 (lane 2) or PC3/OPN (RGA) cells (lane 3). We observed that pretreatment of PC3/OPN cells with a neutralizing antibody against CD44 clearly blocked the OPN-induced activation of MMP-9 (lane 5). PC3 cells secrete active MMP-9 (Figures 1 and 4) although the interaction of both pro- and active MMP-9 was observed on the cell surface. These results suggest that the surface expression of CD44 is required for the activation of MMP-9. Secretion of active MMP-9 by PC3 cells may offer a potential mechanistic explanation for the processes of ECM degradation and cell migration.

The other half of the immunoprecipitate was blotted with streptavidin HRP to detect surface levels of CD44 (Figure 3B). In agreement with the data shown in Figure 2, OPN over expression was associated with an up-regulation of vCD44 on the cell surface (Figure 3B, lane 1) as compared with PC3 cells (lane 2). This was not observed in either PC3/OPN (RGA) (lane 4) cells or in PC3/OPN cells treated with a blocking antibody to CD44 (lane 5). A significant decrease in the surface expression of CD44 was observed in these cells (lanes 4 and 5). The mechanism by which anti-CD44 blocks CD44 surface expression is not well understood. OPN/CD44 interaction was shown to be required for cell migration and CD44 synthesis [6]. Based on this observation, we suggest that the failure of interaction of OPN with CD44 by anti-CD44 may reduce CD44 synthesis and surface expression. Changes in the levels of CD44 expression in PC3/OPN and PC3/OPN (RGA) provide insights into the role of the αvβ3 receptor in this process.

Stripping and reprobing of the above blot with an antibody to MMP-9 demonstrated the levels of MMP-9 interaction with CD44 (Figure 3C). MMP-9 levels corresponded to the surface expression level of CD44 in the indicated cell lines (panel C). Immunoprecipitation with a non-immune serum is shown in lane 3 (B and C). These observations suggest a biochemical pathway in which OPN mediated an increase in MMP-9 activity. This activity may take place via an increase in the surface expression of MMP-9 docking protein CD44 through αvβ3-mediated signaling [22,28]. These data demonstrate a role for CD44 in the surface localization of MMP-9.

### The effects of BPs on MMP- 9 activity, migration, and proliferation of PC3 cells

Zymogram analysis of conditioned media of PC 3 cell lines treated with BPs: Giruado et al. have demonstrated that an amino-bisphosphonate zoledronic acid targets MMP-9 expressing macrophages and angiogenesis to impair cervical carcinogenesis [29]. MMP-9 secretion was dose-dependently down-regulated by clodronate in human monocyte/macrophages [30]. Bisphosphonate reduced the activities of MMP-2 and MMP-9 in PC3 cells [31]. To this end, we investigated whether such inhibitory effects of MMP-9 activity by bisphosphonates would block cell migration and proliferation of PC3 cells. First, conditioned media from the indicated cell lines treated with or without BPs (50 μM each) such as alendronate (AL) and pamidronate (PA) for 48 h (Figure 4) were subjected to gelatin zymography. Consistent with the observation shown by others [31], treatment of the indicated PC3 cell lines with BPs, resulted in the reduction of MMP- 9 activity in all the cell lines. As shown in Figure 1, MMP- 9 activity was greater in PC3/OPN (Figure 4A, lane 5) than in PC 3 (lane 2) or PC3/OPN (RGA) cells (lane 8). The MMP-9 activity observed in alendronate-treated PC3 and PC3/OPN cells (lanes 3 and 6) is comparable to the activity observed in untreated PC3/OPN (RGA) (lane 8) or PC3/SiRNA (Figure 1C, lane 6) cells. It appears that the reduced cellular level of OPN itself has an inhibitory effect comparable to that of bisphosphonate. In the immunoblotting analysis with an antibody to MMP-9 (Figure 4B), no significant difference in the secreted level of MMP-9 protein was observed in the indicated PC3 cell lines treated with (lanes 4–6) or without (lanes 1–3) pamidronate for 48 h. MMP-9 activity but not the secreted level of MMP-9 is affected by BP treatment or OPN expression. The MMP-9 activity in the conditioned medium goes together with the surface expression levels of CD44 (Figure 2) in different PC3 cells. These data indicate that the actions of BPs or αvβ3-signaling involve MMP-9 activity as a principle therapeutic target for the control of cancer cell progression and metastasis.

#### Wound closure assay

We next performed wound closure assay in the presence and absence of BP (Figure 4E and 4F) for 48 h. PC3 and PC3/OPN cells were used for this assay. To determine the functional consequences of MMP-9 on cell migration, we performed this assay in the presence of GM6001, a broad-spectrum inhibitor of MMPs (GM, Figures 4G and 4H) for 48 h. Untreated PC3 (panel C) and PC3/OPN (panel D) cells were used as controls. Consistent with the observation shown in Figure 2, PC3/OPN cells exhibited a significant increase in migration (Figure 4D). The increase in migration was not due to proliferation of cells as mitomycin was used to block proliferation. A significant decrease in the rate of wound closure was observed in PC3 and PC3/OPN treated with either pamidronate (PA; Figures 4E and 4F) or GM6001 (GM; Figures 4G and 4H). The numbers at the bottom of each panel represent the distance (in μm) not healed by the migration of cells.

Cell proliferation assay: We then evaluated the anti-proliferation effect of BPs on PC3 cells. Cells were treated with increasing doses of pamidronate for 48 h. The dotted line in Figure 4D indicates the initial cell number plated at 0 h. A dose dependent decrease in cell growth was observed from 10–100 μM pamidronate (Fig.4 4I). Nevertheless, PA did not induce any apoptosis up to a dose of 100 μM pamidronate for 48 h. The decrease in cell number suggests the anti-proliferation or anti-cancer effect of BPs.

### The effects of BPs on the surface interaction of MMP-9 and CD44

**Figure 5:**
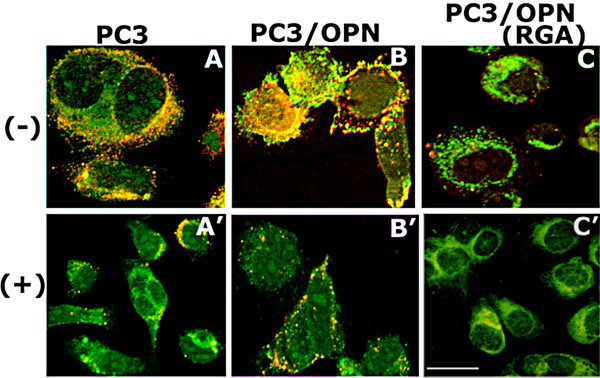
**Immunostaining analysis of the effect of BP on the surface interaction of CD44 and MMP-9 in PC3 cell lines. **Confocal microscopy analysis of distribution of CD44 (red) and MMP-9 (green) in PC3 cell lines treated with (+, A' to C') or without (-, A-C) pamidronate is shown. Yellow color indicates colocalization of proteins on the cell surface. The results represent one of three experiments performed. Scale Bar-50 μm.

Rho signaling pathway is a target for BPs. Rho signaling is essential for CD44 surface expression in osteoclasts [32]. The fact that the expression of Rho GTPase induces clustering of CD44 and MMP-9 in the advancing lamellipodia of endothelial cells [33] prompted us to determine the effects BP on the localization of CD44 and MMP-9 in the indicated PC3 cell lines. The effect of BPs on the surface interaction of CD44 and MMP-9 was analyzed by immunostaining analysis with respective antibodies in cells that were neither fixed with paraformaldehyde nor permeablized with Triton X-100 (Figure 5). We found that PC3 cells express MMP-9 (green) throughout the cytoplasm; however colocalization (yellow) of CD44 (red) and MMP- 9 (green) was observed as clusters on the surface of both PC3 (Figure 5A) and PC3/OPN (Figure 5B) cells. Bisphosphonate treatment significantly reduces colocalization of CD44 and MMP-9 in PC3 as well as in PC3/OPN cells (A' and B'). As shown in Figure 2B, PC3/OPN (RGA) cells had a reduced surface level of CD44 (red). Bisphosphonate untreated (C) or treated (C') PC3/OPN (RGA) cells showed negligible colocalization of CD44 and MMP-9. The diffused distribution of MMP-9 was observed on the surface of these cells. The inhibition level of CD44/MMP-9 interaction in untreated PC3/OPN(RGA) cells (C) is relatively similar to the level observed in PC3 (A') and PC3/OPN cells (B') treated with BP. These results suggest that BP either partially or completely blocks the signaling pathway mediated by integrin αvβ3. MMP-s are Zn-dependent endopeptidases. In an attempt to further elucidate that the interaction of CD44 and MMP-9 on the cell surface is specific, we performed immunostaining analysis with an antibody to human Zip-1 (hZip-1). Human Zip-1 is a cell surface zinc transporter protein that was shown to be expressed ubiquitously on the surface of prostate cancer cells [34]. Immunostaining analysis revealed that the interaction of hZip-1 with either CD44 or MMP-9 was negligible or not at all observed (data not shown). A significant increase in the coclustering of CD44 and MMP-9 on the cell surface of PC3/OPN cells as compared with PC3 cells (Figure 5A and 5B) suggest a plausible mechanism of activation of MMP-9 by CD44.

### Analysis of the effects of osteopontin over-expression on cell morphology and RANKL expression in PC3 cell lines

**Figure 6:**
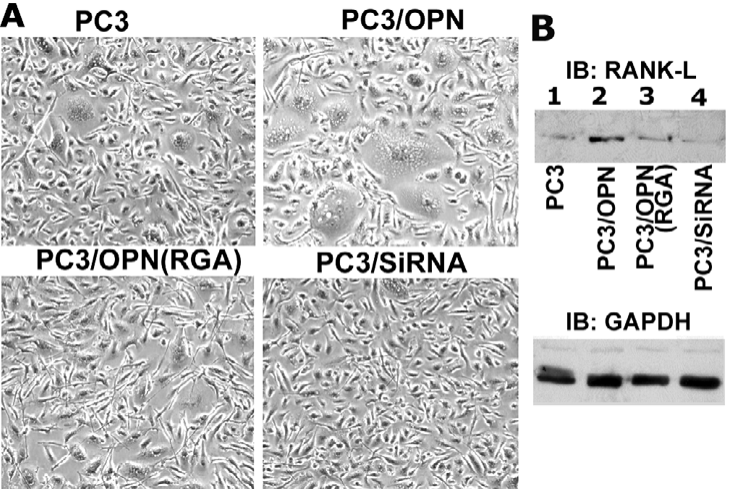
**Analysis of the effects of OPN over expression on cell morphology and RANKL expression in PC3 cell lines. A**. Indicated PC3 cell lines were photographed using a phase contrast microscope. An increase in multinucleated giant cells was observed in PC3/OPN cells (magnification × 200). **B**. Immunoblotting analysis of RANKL expression in PC3 cell lines Immunoblotting analysis in protein lysates made from the indicated cell lines was performed with an antibody to RANKL. PC3/OPN cells express greater level of RANKL that PC3 cells. Results shown are a representation of three independent experiments. Bottom panel shows normalization with GAPDH.

Analysis of morphology of live PC3 cells by phase contrast microscopy: Over-expression of OPN has been shown to augment the occurrence of multinucleated giant cells in pancreatic adenocarcinoma [35] and macrophages of rat glomerulonephritis [36]. These observations prompted investigation on cell morphology in PC3 cell lines that have been used for the above-mentioned studies (Figure 6A). We demonstrated here that the number of multinucleated giant cells was increased in PC3/OPN cells. PC3/RGA cells have multinucleated giant cells at a level comparable to that of PC3 cells. PC3/SiRNA cells failed to display multinucleated giant cells (Figure 6A). In our observations, repeated passaging of PC3/OPN has no effect on the survival of multinucleated giant cells, indicating the genomic stability of this cell line.

#### Analysis of expression of RANKL by immunoblotting analysis

The addition of OPN increased the expression of RANKL and augmented the differentiation of osteoclasts from OPN deficient mice [37]. Since PC3/OPN cells displayed multinucleated giant cells, we presumed that an increased expression of RANKL may be the causative factor for this phenotype (Figure 6B). Immunoblotting analysis with an antibody to RANKL indeed revealed an increased expression of RANKL in PC3/OPN cells (lane 2) as compared with the other PC3 cell lines (lanes 1, 3 and 4). Stripping and reprobing of the same blot with an antibody to GAPDH was used as an internal control for normalization. These observations are in keeping with the observation of Ishi et al., that OPN increased the expression of RANKL [37].

## Discussion

Osteopontin expression has been related to the metastasis of tumor cells [38,39]. OPN functions as a paracrine and autocrine mediator of prostate cancer growth and progression [25]. The biological events that mediate the progression of prostate cancer are not well defined. It was suggested that signaling mediated by integrin αvβ3 facilitated prostate cancer cell progression into bone through their adhesion to and migration on OPN and VN. These are common RGD containing ECM proteins in the bone microenvironment [40]. Our aim was to elucidate the possible role of OPN and its receptor(s)-mediated downstream signals in the progression of prostate cancer cells. The data presented here demonstrates the following: a) OPN over expression increases the interaction of CD44 and MMP-9 on the cell surface; b) integrin and CD44 signaling act as key regulators of MMP- 9 activation; c) the regulation of MMP- 9 occurs through the activation of latent proenzyme (pro MMP-9) on the cell surface; d) OPN/αvβ3-mediated signaling and CD44 expression on the cell surface delineate a likely mechanism in MMP-9 activation and prostate cancer cell migration; e) an increased expression of RANKL in prostate cancer cells provides a plausible additional mechanism in the signaling pathway that leads to CD44 surface expression and MMP-9 activation.

*De novo *expression of CD44 and its variant isoforms has been associated with the aggressive behavior of various tumors [41,42]. Most widely studied CD44 in clinical cancers are probably CD44s, CD44v6, and CD44v3 [43]. CD44v6 and v9 were shown to frequently express in prostate cancer. Our results showed that PC3 cells express multiple high molecular weight isoforms (CD44v) and CD44s core molecule on the cell surface in an OPN/αvβ3 -dependent manner (Figures 2 and 3). Expression of these proteins is increased in PC3/OPN cells. Although, OPN has been shown to be an important ligand for CD44, our data suggests that OPN up-regulated cell invasiveness in an RGD and integrin-dependent manner of CD44 activation rather than having a direct effect on vCD44 in PC3 cells. The vCD44 isoform(s) that has prognostic significance in the metastasis of prostate cancer cells remains to be explored. We suggest that the characterization of vCD44 isoforms necessitates additional studies to unravel its role in cell migration.

We have shown an interaction of MMP-9 with cell surface receptor CD44. Although MMPs interaction with cell adhesion receptors has been observed in different cell systems [19,44-48], the actual mechanism by which they are processed and activated on the cell surface and secreted as an active form is not yet known. OPN expression and MMP-9 activity are linked to prostate cancer cell progression and metastasis [3,39,40]. Active MMP-9 has been found in association with the vCD44 isoform on the invadopodia of breast cancer cells [33,46,49]. We have previously shown the interaction of MMP-2 with cell surface CD44 in human melanoma cells over expressing OPN [10]. In the present study, we show that the surface expression of CD44 and the interaction of CD44 with MMP-9 was reduced in PC3/OPN (RGA) cells as compared with PC3 and PC3/OPN cells (Figures 2, 3, and 5). However, no changes were observed on the surface levels of MMP-9 (Figures 2 and 5). A decrease in MMP-9 interaction with CD44 on the cell surface and secretion of MMP- 9 suggests a role for integrin αvβ3 signaling in the surface expression of CD44.

Cell surface receptors have been shown to provide a docking mechanism for MMPs [10,19,50]. CD44 associates with MMP- 9 on the cell surface of mammary carcinoma and melanoma cells. The inhibition of surface interaction of these proteins by over expression of soluble or truncated CD44 blocked migration and tumor invasion. These observations suggest that CD44/MMP-9 interaction on the cell surface has a role in tumor progression [19]. We show here coclustering of CD44/MMP-9 on the surface is dependent on the expression levels of OPN and CD44 as well as integrin αvβ3 signaling. Pretreatment of PC3/OPN cells with a neutralizing antibody to CD44 competitively blocked the activity of CD44-associated MMP-9 on the surface (Fig. 3). As shown by others [19] and in the present study, CD44 expression on the cell surface may define a mechanism for MMP-9 activation and cancer cell migration.

We have shown secretion of active MMP-9 as well as pro- and active MMP-2 by all the PC3 cells. Integrin αvβ3/CD44 signaling mechanism regulates the secretion of MMP-9 and not MMP-2. It is possible that MMP-2 can be activated by different signaling pathway in prostate cancer cells. Similar to the observation shown here, secretion of an active form of MMP-9 was shown in metastatic breast cancer cells [48]. Secretion of an active form of MMP-9 is more in PC3/OPN cells than PC3/OPN (RGA) or PC3/SiRNA cells (Figures 1 and 4A). However, immunoblotting analysis exhibited equal levels of MMP- 9 protein in the conditioned medium (Figure 4B). Although, a large excess of MMP-9 protein is secreted by all these cell lines, the levels of MMP-9 activity in the conditioned medium mirror the pattern of CD44 surface expression. This indicates that portion of this protein can be secreted as or turned into an inactive form. Lack of pro or intermittent form of MMP-9 in the gelatin zymography supports this conclusion (Figures 1 and 4). The mechanism by which the inactive MMP-9 is secreted needs to be investigated. Secretion of active MMP-9 seems to be dependent on the cooperative interaction between the two cell surface receptors, integrin αvβ3 and CD44.

We show here the inhibitory effect of bisphosphonates (e.g. alendronate and pamidronate) on MMP-9 activity, CD44 surface expression, cell surface CD44/MMP-9 interaction, and migration (Figs. 4 and 5); without increasing apoptosis of cells. The data are consistent with the observations by Bossier et al that BP blocks tumor cell invasion by the inhibition of proteolytic activity of MMPs but not by inducing apoptosis [20]. Alendronate blocked MMPs secretion and bone collagen release by PC3 cells in SCID mice [51]. Recent findings have shown that alendronate and other BPs block the mevalonate pathway, thereby preventing prenylation of small GTPase signaling proteins that are required for both osteoclast function [52,53] and human ovarian cancer cell migration [54]. It has been suggested that Rho A triggers signaling pathways that upregulated the expression of MMP-9 at specific membrane localizations. These localizations may confer a highly invasive phenotype to endothelial cells [33]. We have previously shown that OPN/αvβ3-mediated Rho signaling is required for CD44 surface expression [22]. Based on these findings, we postulate the notion that decreased Rho signaling by BPs could be responsible for the reduced surface expression of CD44, surface interaction of CD44/MMP-9, and cell migration (Figures 4 and 5) in PC3 and PC3/OPN cells.

Bone metastases are a common occurrence in a number of cancers including lung, breast, and prostate. Bone resorption markers were found to be elevated in patients with prostate cancer and bone metastasis [55]. Bisphosphonates are inhibitors of bone resorption mediated by osteoclasts and were shown to reduce bone pain from metastatic prostate cancer [56]. Prostate cancer cells in bone have been shown to secrete factors such as, PTHrP and RANKL [57-59]. It was shown recently that tumor metastatic activities is mediated by OPG/RANK/RANKL/MMP-9 signaling in PC3 cells and is inhibited by genistein [58]. Expression of OPG/RANK/RANKL in cancer cells has been suggested to have a role in the expression and activity of MMP-9 as well as cancer metastasis [60,61].

**Figure 7:**
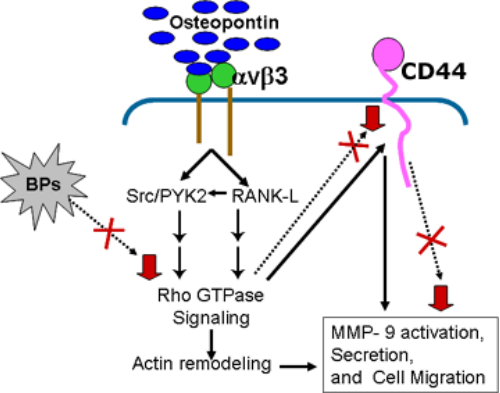
**A schematic representation of osteopontin/αvβ3 signaling in the regulation of prostate cancer cell migration. **PC3 cells bind osteopontin through integrin αvβ3 in an RGD-dependent manner. Integrin αvβ3 increases Rho GTPase activity through down stream signaling pathway that involves RANKL [62] or tyrosine phosphorylation of several signaling molecules such as Src, PYK2, FAK, and p130^Cas ^associated with integrin [10,64]. Rho activation increases CD44 surface expression [10,32] that resulted in the activation of MMP-9. The interaction of CD44 with MMP-9 on the cell surface may have a role in the degradation of ECM to facilitate cell migration. Bisphosphonates (BPs) inhibit CD44 surface interaction with MMP-9 either partially or completely. The signaling pathway and the target molecules that exhibit the inhibitory effects of BP treatment are indicated by dotted arrows and 'X'. A decrease (indicated by inverted red arrow) in CD44 surface expression, MMP-9 interaction with CD44 on the cell surface, MMP-9 secretion, and cell migration was observed in cells treated with BP. PC3/OPN cells treated with BPs reproduce the changes observed in PC3/SiRNA and PC3/OPN (RGA) The possible reason for these effects may be due to reduced αvβ3 -signaling in PC3/OPN (RGA) and PC3/SiRNA cells or targeting of αvβ3-signaling pathway by BPs.

As OPN expression has been shown to increase RANKL expression [37], we analyzed the levels of RANKL in PC3 cell lines. A decrease in the level of RANKL in PC3/OPN (RGA) as compared with PC3/OPN cells suggests that αvβ3 signaling may have a role in the expression of RANKL. Similar to OPN/αvβ3 in the regulation of Rho signaling pathway [22], RANKL also induces Rho signaling pathways in osteoclasts [62]. It is possible that a cumulative increase in Rho activation through αvβ3- and RANKL-mediated down stream signaling pathway may have a direct effect on the surface expression of CD44 in PC3/OPN cells (Figure 7-Scheme). Bisphosphonate was shown to block the mevalonate pathway that inhibits small Rho GTPases [52], which was shown to be crucial for CD44 surface expression [22]. Bisphosphonates also down regulate the expression levels of RANKL in osteosarcoma cells [63]. Finally, one must consider the possibility that the increase in CD44 surface expression, MMP-9 activation, and the migration of prostate cancer cells may occur through multiple down stream signaling pathways that are instigated by αvβ3 receptor.

## Conclusion

Observation in different PC3 cell lines indicate an underlying correlation between the surface levels of CD44 and secretion of active form of MMP- 9 in prostate cancer cells. These results, taken together, suggest some prognostic importance of OPN protein and integrin αvβ3 signaling pathway in the possible vicious action of tumor cells and underlying pathology. The coordinated regulation of proteins such as OPN, αvβ3, RANKL, CD44, and MMP-9 may provide a physiological mechanism for prostate cancer cells to promote migration during metastasis.

## Methods

### Reagents

Antibodies to the standard and variant (V3–V10) CD44 were purchased from Biosource International, Inc. (Camarillo, CA). Antibodies to MMP-2 and MMP-9 were purchased from Chemicon International, Inc. (Temecula, CA). Cy2- and Cy3-conjugated secondary antibodies were purchased from Jackson Immunoresearch Laboratories, Inc. (West Grove, PA). GAPDH antibody was purchased from Abcam, Inc. (Cambridge, MA). Biotin (EZ-link Sulfo-NHS-LC Biotin), streptavidin-HRP, and ECL- reagent were bought from Pierce (Rockford, IL).

### cDNA constructs, cell lines and culture

Mutation in the integrin-binding motif (Arg-Gly-Asp [RGD] Δ Arg-Gly-Ala [RGA]) of human OPN [21] was generated using altered sites II *in vitro *mutagenesis system (Promega, Madison WI). Prostate cancer epithelial cells (PC3, CRL-1435; ATCC; Manassas, VA) were transfected with full-length (PC3/OPN), mutant OPN (PC3/OPN [RGA]) in pCEP4 vector, and vector without insert (pCEP4) with use of Lipofectamine 2000 (Invitrogen, USA) following the manufacturer's instructions.

The OPN SiRNA expression vector was generated using Seqwright DNA Technology Services (Houston, TX). A minimum of four SiRNA constructs for the OPN gene were generated to allow selection of one that expresses an optimal silencing effect. Scrambled RNA construct was used as a control for SiRNA constructs. For each construct, two oligonucleotides that encode sense and antisense sequences separated by a short spacer region (approximately 5–7 bp, forming the loop structure) were designed and synthesized. Location of the target sequence for the constructs are 126–146, 383–403, 649–669, 869–889 in OPN cDNA. PC3 cells were transfected with the SiRNA constructs and pSilencer 4.1-CMV neo vector (as vector control) using a silencer SiRNA transfection kit (Ambion, Austin, TX).

Individual clones (about 15–20) were isolated following transfection of PC3 cells with the above-mentioned OPN constructs. PC3 cells were transfected with use of Lipofectamine 2000 (Invitrogen, USA) following the manufacturer's instructions. After 48 h transfection, cells were maintained in medium that contained different concentrations of G418 sulfate, for selection. Cells were kept in 400 μg/ml G418 for 3 d followed by 200 μg/ml G418 sulfate for 7–10 days and continued in 100 μg/ml G418 for 20 days. After selection, G418-resistant clones were observed as cell clusters. Individual clones were isolated for each construct in 24-well tissue culture plates using cloning filters (Sigma). Once the small cultures grew to near confluency, they were transferred to 60 mm tissue culture dishes with RPMI medium containing 10% FBS. Clones were maintained individually for each construct. OPN expression levels were determined by immunoblotting analysis with a polyclonal antibody to OPN (generated in Sigma-GenoSys). Four clones (C1–C4) that express elevated levels of full-length and mutated OPN (RGDΔRGA) were selected. To reduce the endogenous levels of OPN, four different SiRNA constructs were transfected into PC3 cells. PC3 cells transfected with respective vectors and untransfected PC3 cells were used as controls. Maximum decrease was observed with a SiRNA construct targeted to sequence 383–403 in human OPN cDNA. Individual clones (C1–C5) that exhibited maximum reduction in endogenous OPN levels were generated from this cell line. PC3 clones that express the highest levels of OPN (full length and mutant) and a maximum reduction in the endogenous levels of OPN were used for all the experiments described here. These clones were designated as PC3/OPN, PC3/OPN (RGA) and PC3/SiRNA.

### Cell culture and Treatments

PC3 cell lines were cultured in Roswell Park Memorial Institute-1640 (RPMI-1640) media containing 10% FBS at 37°C (Gibco-BRL, Bethesda, MA). Bisphosphonates (BPs), such as alendronate and pamidronate were used for experiments. Stocks (1 mM) were made in PBS or sterile H_2_O. Prostate cancer cells were treated with BPs to a final concentration of 50 μM for 48 h at 37°C. Some cultures were treated either with the blocking antibody to CD44 (30–50 μg/ml; BioSource AHS 4418) or GM6001 (15 μM at 37°C) for 48 h. Following various treatments, cells were washed three times with cold PBS and lysed with RIPA lysis buffer as described previously [22]. Some cultures were used for immunostaining analysis as described below. For zymogram analysis, cells were kept in serum-free RPMI medium for 24–48 h in the presence and absence of various treatments. Conditioned media collected from various PC3 cell lines were subjected to gelatin zymography as described below and previously [10].

### Immunostaining

Surface localization of CD44 and MMP-9 was determined in cells that were not permeablized with paraformaldehyde or fixed with Triton X-100 by immunostaining with antibodies to variant CD44 (V3–V10) and MMP-9 as described previously [22].

### Cell surface labeling by biotinylation and Immunoblotting analysis

After various treatments, cells were labeled with NHS- biotin according to the manufacturer's guidelines (Pierce, Rockford, IL). Briefly, cells were incubated with 0.5 mg/ml biotin for 30–40 min. at 4°C and washed three times with cold PBS. Cells were lysed with RIPA lysis buffer as described previously [22]. Equal amounts of protein lysates were used for immunoprecipitation with an antibody to CD44 or MMP-9. Immunopecipitation of two proteins at the same time with two different antibodies (e.g. CD44 and actin as shown in Figure 2) was also performed. The immune complexes were adsorbed onto Protein-A sepharose beads or streptavidin agarose. Streptavidin agarose precipitates the biotinylated CD44 protein from the cell surface. The immune complexes adsorbed onto streptavidin agarose were washed three times with cold PBS and incubated with sample buffer with no reducing agent (β ME or DTT) for 10–15 min. at room temperature (RT). Zymogram analysis was performed as described below. MMP-9 activity associated with cell surface CD44 was analyzed by gelatin zymography as described below. The immune complexes adsorbed onto streptavidin agarose were also used for immunoblotting analysis with streptavidin-HRP to detect the cell surface levels of CD44. The immune complexes adsorbed to Protein-A sepharose beads were immunoblotted with streptavidin HRP to detect the surface level of CD44 or with a primary antibody of interest (e.g. actin). Immunoblotting with an actin antibody was used as a loading control. Prior to loading on SDS-PAGE, these samples were boiled with loading buffer containing βME. SDS-PAGE analysis and immunoblotting was performed as described previously [22,23]. Protein bands were visualized by chemiluminescence using the ECL-kit (Pierce, Rockford, IL).

### Gelatin zymography

Conditioned media collected from various PC3 cell lines were concentrated approximately 10-fold) with a centricon concentrator (Amicon, Beverly, MA). Ten micrograms of concentrated media protein was diluted to 20 μl in cold PBS. Immune complex made with CD44 (V3–V10) antibody, as described above, was also used for this analysis. Samples were mixed with SDS gel loading buffer with no reducing agent (βME or DTT) and incubated at RT for 10–15 min to dissociate immune complexes. SDS-PAGE containing 0.1% gelatin was used for electrophoresis. Samples were loaded without heating with sample buffer. After electrophoresis, gels were incubated overnight in a buffer containing 50 mM Tris-HCl, pH 7.6, 5 mM CaCl_2_, 1 μM ZnCl_2_, and 1% Triton X-100. Triton was used to remove SDS from the gel. Gels were then stained with coomassie brilliant blue for 2–3 h and destained with 7% acetic acid or water. Gelatinolytic activity was detected as clear bands in the background of blue staining [24].

### Analysis of cell migration by wound closure assay

Wound closure assay was performed as described previously [10]. Cells were grown in 35 mm culture plates (Falcon) to near-confluent level in RPMI medium containing 10% fetal bovine serum (FBS; Cellgro). Multiple uniform streaks (~50 μm in width) were made on the monolayer culture with 10 μl pipette tips. The cells were immediately washed with RPMI-medium with 10% FBS to remove detached cells. Mitomycin (5 μg/ml; Sigma) was used in the medium to inhibit proliferation of cancer cells. Hence, the observed increase in PC3 cells is not due to increase in proliferation of cells. The experiment was performed with GM6001 (15 μM) and bisphosphonates (50 μM). The cell migration was monitored for 48 h, and pictures were taken at 0 h and 48 h time points with a digital SPOT camera attached to an inverted Nikon phase contrast microscope. Six to eight fields were analyzed, and the mean percentage of wound area covered by cells was calculated. The amount of wound closure remaining between the original wound widths was traced with SPOT Advanced 3.0.4 image software (Diagnostic Instruments, Inc.). The distance between the migratory cells in the wound area at a 90° angle to the wound margin generated at 0 h was measured using the same program. The distance of wound width was expressed in microns before and after migration. Following each experiment, cell viability was confirmed by trypan blue dye exclusion assay. Floating cells were marked with black arrows (Figures 2 and 4). Statistical analysis was performed as described below.

### Cell proliferation assay

PC3 cells were plated in 6-well chambers at a confluence of 2.5 × 10^4 ^cells per well in RPMI-1640 medium containing 10% FBS at 37°C for 18 hours. Cells were then treated with increasing doses of pamidronate as indicated. Cells treated with PBS were used as controls. Incubation was continued for 48 h. Cells were counted using a Neubauer's chamber at the end of 48 hours.

### Statistical analysis

All values presented as mean ± SEM. A value of p < 0.05 was considered significant. Statistical significance was determined by analysis of variance (ANOVA) with the Bonferonni corrections (Instat for IBM; Graphpad software).

## List of abbreviations

OPN, osteopontin; MMP-9, matrix metalloproteinase 9; αvβ3, vitronectin receptor; variant CD44, vCD44; RGD, amino acid sequences such as, Arginine(R), Glycine (G) and Aspartic acid (D). It is also known as integrin binding motif; RGA, amino acid sequences such as Arginine (R), Glycine (G) and Alanine (A); PC3/OPN, PC3 cells over expressing OPN; PC3/OPN (RGA), PC3 cells over expressing mutated OPN in integrin binding motif, RANKL, receptor activator of NF kappa B ligand; BP, bisphosphonate

## Competing interests

'The author(s) declare that they have no competing interests'.

## Authors' contributions

BD participated in the design of the study as well as carried out the biochemical and cell biological studies. MR provided bisphosphonates. MAC conceived of the study, participated in its design, and drafted the manuscript. All authors read and approved the final version of the manuscript.
